# Platelet Biorheology and Mechanobiology in Thrombosis and Hemostasis: Perspectives from Multiscale Computation

**DOI:** 10.3390/ijms25094800

**Published:** 2024-04-27

**Authors:** Rukiye Tuna, Wenjuan Yi, Esmeralda Crespo Cruz, JP Romero, Yi Ren, Jingjiao Guan, Yan Li, Yuefan Deng, Danny Bluestein, Zixiang Leonardo Liu, Jawaad Sheriff

**Affiliations:** 1 Department of Chemical & Biomedical Engineering, FAMU-FSU College of Engineering, Tallahassee, FL 32310, USA; rtuna@eng.famu.fsu.edu (R.T.); eec19f@fsu.edu (E.C.C.); leo.liu@eng.famu.fsu.edu (Z.L.L.); 2 Department of Biomedical Sciences, College of Medicine, Florida State University, Tallahassee, FL 32304, USA; 3 Institute for Successful Longevity, Florida State University, Tallahassee, FL 32304, USA; 4 Department of Applied Mathematics and Statistics, Stony Brook University, Stony Brook, NY 11794, USA; 5 Department of Biomedical Engineering, Stony Brook University, Stony Brook, NY 11794, USA; danny.bluestein@stonybrook.edu

**Keywords:** von Willebrand factor, platelet margination, platelet adhesion, shear-induced platelet aggregation, platelet mechanobiology, biorheology, platelet activation, thrombosis

## Abstract

Thrombosis is the pathological clot formation under abnormal hemodynamic conditions, which can result in vascular obstruction, causing ischemic strokes and myocardial infarction. Thrombus growth under moderate to low shear (<1000 s^−1^) relies on platelet activation and coagulation. Thrombosis at elevated high shear rates (>10,000 s^−1^) is predominantly driven by unactivated platelet binding and aggregating mediated by von Willebrand factor (VWF), while platelet activation and coagulation are secondary in supporting and reinforcing the thrombus. Given the molecular and cellular level information it can access, multiscale computational modeling informed by biology can provide new pathophysiological mechanisms that are otherwise not accessible experimentally, holding promise for novel first-principle-based therapeutics. In this review, we summarize the key aspects of platelet biorheology and mechanobiology, focusing on the molecular and cellular scale events and how they build up to thrombosis through platelet adhesion and aggregation in the presence or absence of platelet activation. In particular, we highlight recent advancements in multiscale modeling of platelet biorheology and mechanobiology and how they can lead to the better prediction and quantification of thrombus formation, exemplifying the exciting paradigm of digital medicine.

## 1. Introduction

Thrombosis in vascular disease is triggered by the interaction of blood constituents with an injured blood vessel wall and non-physiologic flow patterns characteristic of cardiovascular pathologies. The shear rates and stresses to which blood constituents are exposed are dependent on the geometry of the blood vessel and its disease state (Table 1). The process for arterial thrombus formation in a high-shear environment differs from that for low-shear thrombosis (such as deep venous thrombosis), mostly governed by the Virchow Triad, which postulates that thrombosis is regulated by three components: endothelial wall damage, stasis of blood flow, and hypercoagulability [1]. Thrombus formation at a high shear rate is mediated by an alternative triad, a combination of von Willebrand factor (VWF), a high shear environment, and a thrombotic surface (e.g., subendothelial collagen matrix) [2,3,4,5]. Recent studies have shown that VWF plays a vital role in high-shear thrombus formation [3,4,6] through a process called shear-induced platelet aggregation (SIPA), which entails the rapid binding and entanglement of VWF polymers with inactivated platelets under elevated high shear rates (>10,000 s^−1^) [7]. The SIPA process involves three sequential stages [3]: (1) VWF elongation, (2) platelet aggregation, and (3) agglomerate capture. Stage 1 involves the conformational change in VWF from a globular to an elongated state that reveals many A1 domains to bind platelet glycoprotein Ibα (GPIbα) receptors in the flow. Stage 2 is associated with forming platelet agglomerates resulting from GPIbα-VWF A1 binding. In this stage, GPIbα-VWF A1 interactions lead to high-shear platelet tethering and mechanotransduction- and biochemical mediator-facilitated platelet activation [8,9,10,11]. In Stage 3, the platelet agglomerate approaches the immobilized VWF surface, rolls, and translates on the surface until fully captured before platelet activation further reinforces the accumulation of platelets via $\alpha_{IIb}\beta_{3}$-fibrinogen binding.

The disparate spatio-temporal scales between molecular-level inter- and intra-platelet events to macroscopic transport in blood flow pose a major modeling and computational challenge, which requires the careful selection of models of specific scales or even the integration of multiple numerical techniques suitable for distinct scales. Molecular dynamics (MD) allows for atomistic interactions (i.e., GPIb-A1 binding [13], integrin $\alpha_{IIb}\beta_{3}$-fibrinogen binding [14], etc.) with high fidelity. However, MD is computationally cost-prohibitive and is limited to nanometer-length-scale events that occur mostly within nanoseconds or microseconds. Lattice-Boltzmann and Dissipative Particle Dynamics (DPD) as mesoscopic methods can be used to model blood as a polydisperse particulate fluid, promising to address micro- to millisecond scale events at which flow-mediated binding often occurs, and which are critical for macroscopic growth or embolization [15,16,17,18]. Continuum approaches represent platelet accumulation or coagulation by field equations and moving boundaries, which permits shape change with interfaces of different properties and time scales [19,20]. Continuum systems, however, are limited to macroscopic fluid scales that homogenize the microscale details and require careful closure of the system of equations through constitutive relations [21,22].

The following sections describe the various approaches to modeling platelet mechanobiology, its role in thrombus formation, and the innovative multiscale approaches used to elucidate the complex nature of thrombosis. These sections are divided by the major (not necessarily sequential) steps in thrombus formation: platelet margination, adhesion, activation, and aggregation. This is followed by a brief description of recent works on using artificial intelligence (AI) to enhance the multiscale modeling of platelet mechanobiology and a perspective on the future of this field.

## 2. Platelet Margination

**Figure 1:**
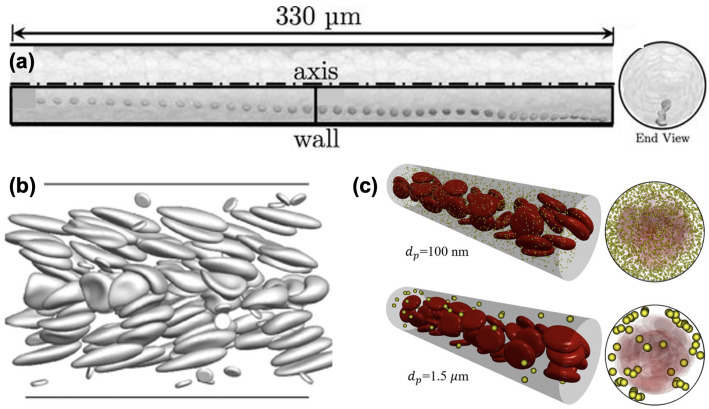
(**a**) Platelet imagination in a 3-D microvessel (adapted with permission from Reasor et al. [26]). (**b**) Snapshots of platelet imagination and flipping near the wall in a 2-D channel flow (adapted with permission from Zhao and Shaqfeh [27]). (**c**) Margination occurs only for microscale particles, not for nanoscale particles. (adapted with permission from Liu et al. [28]).

Platelet margination is a physiological phenomenon that occurs as platelets migrate toward the vessel wall and become retained in the cell-free layer (CFL), where red blood cells (RBCs) are hydrodynamically depleted [23,24]. Margination could enhance the near-wall platelet concentration [25] and has been found to support the attachment of platelets and subsequent platelet clot formation [26] (Figure 1a). Over the past decade, computational particulate flow techniques have emerged to quantify the rheological nature of margination at cellular scales.

Crowl and Fogelson [24] examined how platelet margination varies with shear rate, hematocrit, and platelet size through a two-dimensional (2D) lattice Boltzmann-immersed boundary method. Using a three-dimensional (3D) boundary integral method, Zhao and Shaqfeh [27], Zhao et al. [29] showed that particle margination correlates with the fluctuations of velocity fields mediated by RBC interactions in the suspension to understand the platelet margination mechanism at vessel walls (Figure 1b). Combining theory and computation, Kumar and Graham [30,31] attributed the margination and segregation of cells to the heterogeneous pair collision due to disparities in cell rigidity. Using a 3D lattice Boltzmann-based method, Reasor et al. [26] found that the margination rate increases with hematocrit and spherical particles marginate faster than disk-shaped platelets. Combining 3D simulations and scaling analysis, Mehrabadi et al. [23] showed that the margination length scales cubically with vessel size and is independent of shear rate. They estimated that it would take ∼20 mm for platelets to fully marginate within a 40 μm diameter vessel. Using a multiscale particulate blood flow solver, Liu et al. [28,32] demonstrated that particles need to be at least 1 μm in size to give rise to margination (Figure 1c). Furthermore, the same group quantified the full diffusion rate tensor of platelets transported in blood flows, providing a more accurate constitutive relation for continuum-level computational modeling of platelet transport in blood flows [33]. It is now elucidated that platelet margination is primarily driven by the biorheological effect of blood as a complex fluid. Given the presence of a margination distance [23], more studies are needed to have a better understanding of how non-trivial vascular geometries such as bifurcation, curved vessels and stenosis alter the transient margination behavior and the thrombogenicity.

## 3. Platelet Adhesion

**Figure 2:**
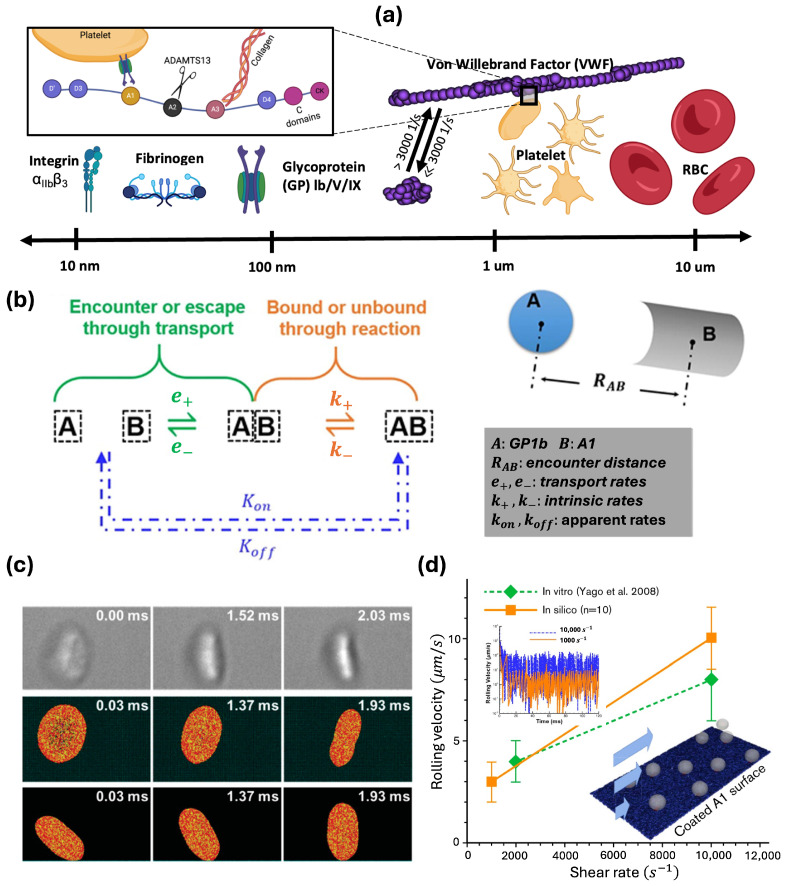
(**a**) Cellular and molecular components related to platelet adhesion ranging from nano (nm) to micron (μm) scales (created with BioRender.com). (**b**) Binding kinetics of GPIb-VWF A1 in flow can be decoupled into transport and reaction components (adapted with permission from Liu et al., 2021 [7]). (**c**) Snapshots of single platelet flipping motion correlating with in vitro observations (adapted with permission from Wang et al., 2023 [47]). (**d**) Platelet rolling velocity measured based on a multiscale computational model built incorporating molecular and cellular components and their binding kinetics [41] (adapted with permission from Liu et al., 2022 [3]).

Platelet adhesion to the vessel wall is primarily mediated by VWF and fibrinogen, two of the most physiologically relevant ligands that support thrombosis and hemostasis [34]. Platelet adhesion to fibrinogen is through the binding of integrin $\alpha_{IIb}\beta_{3}$ to fibrinogen molecules, while adhesion to VWF is through the binding of platelet receptor GPIbα to the VWF-A1 domain. While both support platelet adhesion, the two ligand-receptor pairs show distinct biomechanistic characteristics. As shown by Savage et al. [35,36], $\alpha_{IIb}\beta_{3}$-fibrinogen binding is efficient at wall shear rates of 50–500 s^−1^, while the GPIbα-VWF A1 binding is supported by pathologically high shear rates above 6000 s^−1^. For shear rates from 500 s^−1^ to 6000 s^−1^, the two binding modes play complementary roles in supporting immediate surface arrest or marked reduction in flow velocity, respectively, with the efficiency of GPIbα-VWF A1 increasing with shear [35]. In addition, $\alpha_{IIb}\beta_{3}$-fibrinogen binding relies on platelet activation and can be irreversible, while GPIbα-VWF A1 binding is reversible and independent of platelet activation [37]. Unlike fibrinogen, VWF is a multimer polymer chain that can elongate to expose hundreds of binding sites and extend to tens to hundreds of microns, further enhancing binding events through multivalency. The disparate length scales of all the molecular and cellular players pertinent to platelet binding and adhesion are summarized and highlighted in Figure 2a.

### 3.1. Recent Progress on Quantifying Shear-Dependent Binding Kinetics

Single-molecule experimental assays have enabled direct measurement of platelet binding kinetic rates. Fu et al. [38] measured the apparent on rates of GPIbα-VWF A1 to be above 10^6^ M^−1^s^−1^ using fluorescence single-molecule microscopy. Their measurement is consistent with the earlier work by Wellings and Ku [39], where the GPIbα-VWF A1 bond on (formation) rate is estimated to range from 10^5^ to 10^9^ M^−1^s^−1^ to support firm platelet adhesion under elevated high shear. Combining apparent on-rate measurements and kinetic theory, Liu et al. [7] derived the intrinsic on-rate of GPIbα-VWF A1 to be around 10^5^ s^−1^, which means it only takes ∼10 μs to form a single bond as long as GPIbα and VWF A1 molecules are sufficiently close. In contrast, the binding of fibrinogen to eptifibatide-primed $\alpha_{IIb}\beta_{3}$ exhibits an on rate of ∼10^4^ M^−1^s^−1^ [40], which is approximately two orders of magnitude lower than the GPIbα-VWF A1 on rates. Besides on rates, the off rates, the reciprocal of bond lifetime, of GPIbα-VWF A1 and $\alpha_{IIb}\beta_{3}$-fibrinogen bonds have also been measured [41,42,43] to be 1–100^−1^, where higher loading forces lead to higher off rates (i.e., shorter bond lifetime). Therefore, the superior effectiveness of the GPIbα-VWF A1 bond compared to the $\alpha_{IIb}\beta_{3}$-fibrinogen bond for high shear platelet attachment is attributed to the ultra-high on-rates of GPIbα-VWF A1 bond that can form within ∼10 μs.

### 3.2. Recent Progress in Multiscale Modelling of Platelet Adhesion

Computational models have been developed to elucidate the rate and pattern of platelet adhesion dynamics. Pioneered by Hammer et al. [44,45], earlier work focused on single-cell adhesion dynamics. Mody et al. [46] established a 2D analytical model and studied the motion pattern of flipping and adhering platelets. Wang et al. [47] utilized a DPD-based model combined with a modified Morse potential (simulating non-bonded electrostatic interactions) to study shear-mediated platelet flipping and adhesion onto an effective VWF surface, showing good correlation with their microchannel perfusion experiments of platelet flipping motion in physiological flow conditions (Figure 2c). While these models can capture the platelet dynamics influenced by the platelet discoid shape and shear transport, they often simplify the effect of molecule components (especially the ligands) through effective kinetics modeling.

More recently, multiscale models have been developed to explicitly incorporate molecular components and cellular information. Liu et al. [7] proposed a first principles-based computational model to simulate platelet adhesion to VWF multimers. The model captures the VWF conformational change and the corresponding platelet dynamics under various shear rates. Moreover, they developed a stochastic binding model based on transport-independent intrinsic rates derived from single-molecule kinetics measurements and classical kinetic theory (Figure 2b). Since the model is built from a bottom-up approach, no empirical tuning was found to be necessary to match existing experiments. This was confirmed by the platelet rolling velocity captured by the in silico model matching well with the in vitro results shown in Figure 2d [3]. Recently, Belyaev et al. [48] also developed a similar model, where GPIbα-VWF A1 binding was modeled through Morse potential with model parameters tuned to match a specific set of experiments.

## 4. Platelet Activation

Platelet activation is defined by morphological and structural changes, membrane phospholipid scrambling and procoagulant activity, activation of surface integrins, initiation of internal signaling pathways, granular secretion, and signaling between surface receptors and ligands that lead to adhesion and aggregation [49,50,51]. Platelet activation can occur after adhesion to the injured endothelial wall or growing thrombus, requires soluble agonists [52], high fluid shear exposure for extended durations, and the interaction of GPIbα with VWF immobilized on the damaged blood vessel wall [53] to initiate mechanotransduction effects. Platelet activation can also occur in pathological stenosis or blood-recirculating cardiovascular devices through shear-induced platelet activation (SIPAct) even with little agonist stimulation [51,54,55].

### 4.1. Shear-Induced Platelet Activation through Mechanotransduction

**Figure 3:**
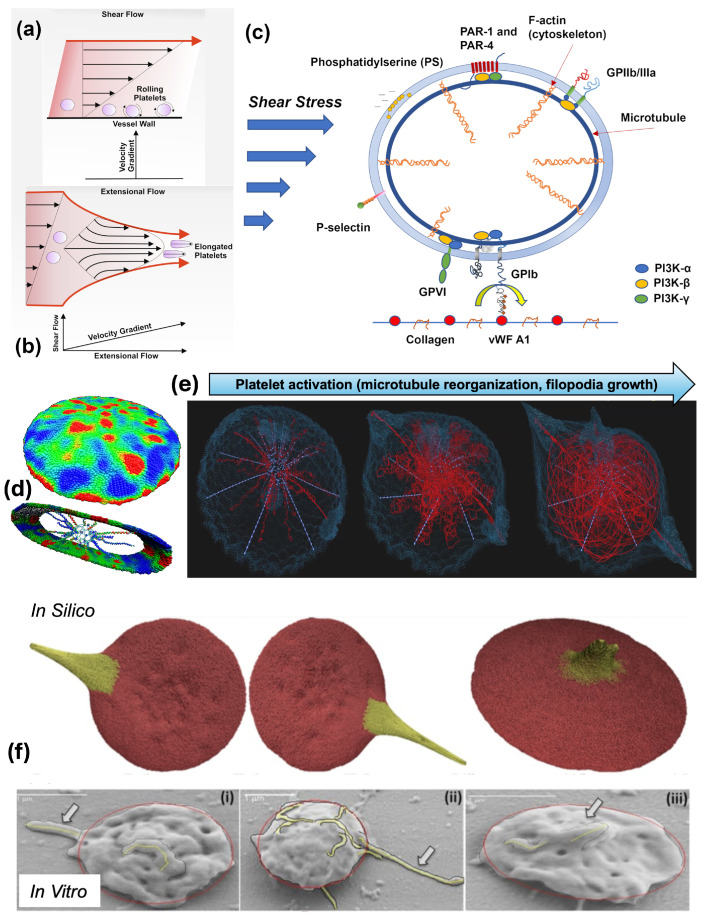
(**a**) Platelets experience wall shear stress as they move through layers of flowing blood and roll along the blood vessel wall (adapted with permission from Zainal Abidin et al., 2023 [107]). (**b**) Platelets elongate as they experience extensional stresses due to fluid acceleration parallel to the wall, particularly in areas of stenoses (adapted with permission from Zainal Abidin et al., 2023 [107]). (**c**) Upstream and downstream participants are involved in shear-mediated platelet mechanotransduction, with PI3K as the primary driver. (**d**) Stress distribution on the platelet membrane and the actin filaments modeled using the hybrid coarse-grained molecular dynamics (CGMD) method. (**e**) The simulation directly captures microtubule reorganization that supports filopodia growth, all occurring during the mechanotransduction-induced platelet activation process. (**f**) CGMD model of platelet activation-induced filopodia formation in comparison with experimental observations: (**i**) scanning electron microscopy image after exposure to 1 dyne/cm^2^ for 4 min, (**ii**) 70 dyne/cm^2^ for 4 min, and (**iii**) 70 dyne/cm^2^ for 1 min (adapted with permission from Pothapragada et al., 2015 [105]).

Platelet function in response to fluid shear stress has been extensively studied for a few decades [56,57,58]. Most approaches have identified the GPIbα-A1 interaction under flow conditions as the primary driver of mechanotransduction [59], where mechanical cues from the surrounding environment are transmitted through the rolling platelet (Figure 3a) and result in structural and biochemical changes. Under pathological high shear flows (i.e., severe stenosis) or elevated high shear conditions in blood-contacting devices (e.g., in left ventricular assist devices), platelet activation can also be mechanically augmented with limited GPIbα-A1 interactions seeing the presence of VWF cleavage [54,60,61]. These mechanical mechanisms of SIPAct may include additive membrane damage (or stress accumulation) [62,63], activation of shear-sensitive channels and pores such as Piezo1 and Pannexin-1 (Panx1) [64,65,66], and outside-in signaling via a range of transducers other than GPIbα and GPIIb-IIIa (integrin $\alpha_{IIb}\beta_{3}$) [55,67]. Piezo1 is a mechanosensitive Ca^2+^ permeable cation channel that may contribute to thrombus formation by promoting Ca^2+^ influx under arterial shear [66], senses supraphysiological flow gradients that generate extensional strain leading to deformation in the platelet structure [68] (Figure 3b), and upregulates $\alpha_{IIb}\beta_{3}$ signaling and promotes aggregation in hypertensive mice [69]. Panx1 amplifies the Ca^2+^ signal from Piezo1 to P2X channels [68]. Platelets adhered to a growing thrombus undergo sustained calcium oscillations, in turn inducing a rapid increase in calcium flux in freely translocating platelets tethered to adherent platelets [11].

Phosphoinositide 3-kinase (PI3K) functions as a hub in mechanotransduction and plays a pivotal role in mechanosensing tension, stretching, and compression of the plasma membrane in a variety of cell types [70]. The PI3K/Akt signaling pathway plays a critical role in platelet mechanotransduction [71], regulating VWF A1-GPIbα interaction, intracellular calcium mobilization, $\alpha_{IIb}\beta_{3}$ activation, adhesion, and thrombus growth [72] (Figure 3c). PI3K generates 3-phosphoinositides, involved in all contexts of platelet activation and integrin function [73], and is a critical transmitter of multiple signaling pathways activated by receptor tyrosine kinases (RTK), G-protein coupled receptors (GPCRs), glycoproteins and integrins [74]. The Class I PI3K p110β isoform, which plays a prominent role in structural modeling and motility, has been identified as a potential antithrombotic therapy target [75], and while it is not required for thrombus growth under physiological conditions, it is necessary for thrombus stability at high shear stress [76]. The PI3K C2α isoform has been identified as essential for Piezo1 activation and may participate by stiffening the cortical cytoskeleton that resists platelet deformation or altering the lipid bilayer membrane composition [68]. Altering platelet mechanical stiffness can also reduce platelet activation under elevated high shear [55].

### 4.2. Resting and Activated Platelet Morphology under Flow Conditions

Resting or quiescent platelets are generally approximated as oblate spheroids [77], commonly adopted by computational models [78,79]. Submembrane cortical structural elements involved in shear-induced platelet activation include actin, myosin, spectrin and intermediate filaments [80], providing tension to the platelet surface and allowing the lipid bilayer membrane to “wrinkle” [81]. During activation and spreading, the bilayer unfolds to a larger surface area and provides a buffer for changes in surface membrane tension, effectively dampening platelet activation resulting from rapid blood flow fluctuations [82]. The microtubule marginal band generates the platelet discoid structure and consists of dynamic microtubules [83,84]. When the platelet is activated, the microtubule coil disassembles and reduces in diameter [84] and plays a diminished role in platelet function [85]. The actin cytoskeleton supports the platelet’s discoid structure and actively aids in platelet spreading [86]. Compressed spectrin-rich networks sandwiched between the membrane and cytoskeleton intersperse GPIb-IX-actin binding protein complexes connected to filamentous actin (F-actin) radially projecting from a central cross-linked F-actin core [86]. Upon activation, soluble F-actin polymerizes into an average of 2000 filaments, each approximately 1.1 μm long, promoting the formation of platelet pseudopods [86]. Upon activation, actin is fragmented, and the platelet assumes a spherical form [80]. The spectrin network then swells and allows the protrusion of spindle-like filopods or sheet-like lamellipods with additional actin-assembling signals [87].

Under pathological flow conditions, such as those found in arterial stenoses, platelets transform into spherical shapes, show extended pseudopods, and have occasional organelle centralization [88]. Integrin $\alpha_{IIb}\beta_{3}$ receptors redistribute and relocate to the pseudopod extremities, but platelets remain in a state of reversible activation [88]. Platelets in stroke patients show significant cytoskeletal rearrangement [89]. Under hypershear conditions, platelets exhibit more dramatic shape change, with some platelets exhibiting severe damage characterized by leaky membranes and total breakdown of the microtubule band and actin cytoskeleton [90,91]. Membrane stretching associated with elongational stresses in the descending aorta during diastole may allow a conformational change in the platelet receptors, increasing ligand binding affinity and allowing increased ion permeability [92]. Hypershear stresses and short exposure times found in cardiovascular devices such as ventricular assist devices promote $\alpha_{IIb}\beta_{3}$, GPIbα, and GPVI receptor shedding, additionally promoting bleeding complications [93,94,95], and procoagulant microparticles are formed and released from the platelet surface [96].

### 4.3. Recent Progress of Multiscale Modelling of Shear-Induced Platelet Activation

Early multiscale models of shear-induced platelet activation simplified platelets as an ensemble of bound particles with an enclosing membrane or continuous solid resembling a rigid ellipsoid shape, neglecting molecular intraplatelet constituents and membrane deformability [46,97,98,99,100,101]. Two of these early models were adapted to include subcellular elements (SCE) [101,102] to represent the cytoskeletal network and continuum description of the lipid bilayer, thereby enabling modeling of platelet motion and deformation in flowing blood, described by a lattice-Boltzmann approach, as well as variability in the platelet stiffness [102]. Zhang et al. [103] developed a coupled approach where DPD describes the top-scale viscous fluid flow. The bottom-scale deformable platelet utilizes a CGMD approach to build a molecular model of platelets (Figure 3d). This CGMD model consists of approximately 140,000 particles representing viscoelastic bilayer membrane, functional actin cytoskeleton consisting of α-helix filaments, supporting actin core, and cytoplasm. The particles interact using bonded spring forces and non-bonded Lennard–Jones electrostatic potentials [103,104]. Using this approach, Pothapragada simulated platelet shape change and filopodia formation under shear stresses up to 70 dyne/cm^2^, in good agreement with in vitro experiments (Figure 3f) [105]. Zhang et al. showed that this model allows regional mapping of hemodynamic stresses encountered in flowing blood to the bilayer membrane and actin cytoskeletal structure, thereby identifying locations on and within the platelet likely to undergo mechanotransduction (Figure 3e) [104], as well as predict locations where filopodia are likely to form [106]. The platelet CGMD model was updated to include the microtubule function, where extension of filopodia are anchored at the filamentous core and allowed to coil around the submembrane platelet periphery as filopodia protrude from the platelet [106].

## 5. Platelet Aggregation

**Figure 4:**
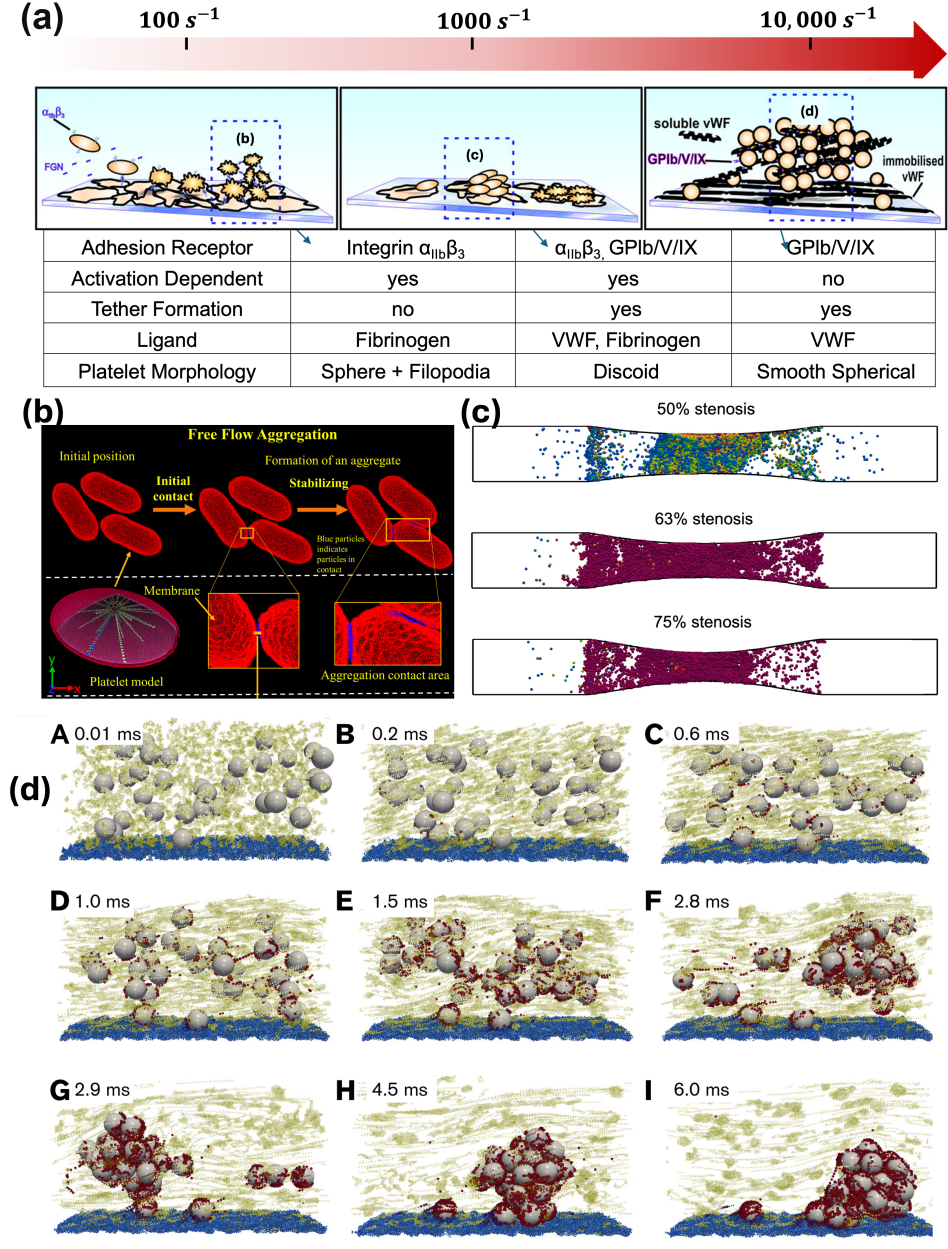
(**a**) Different pathways of platelet aggregation under various levels of shear rates (adapted with permission from Maxwell et al. [108]). (**b**) Simulated aggregation of three adjacent platelets through a DPD-CGMD coupled multiscale method. (adapted with permission from Gupta et al. [112]). (**c**) Snapshots of platelet aggregate formation at the wall due to agonist-induced platelet activation (adapted with permission from Shankar et al. [115]). (**d**) Activation-independent platelet aggregation occurs in less than 10 ms (adapted with permission from Liu et al. [3]).

Platelet aggregation can be supported through different pathways depending on the levels of shear rates [6,108], as demonstrated in Figure 4a. At low shear rates (100 to 1000 s^−1^), platelet aggregation relies on platelet activation as a prerequisite to activate the integrin $\alpha_{IIb}\beta_{3}$ adhesion to fibrinogen [6,35,36,108,109] supported by platelet morphological changes, such as spheroid structure and filopodia formation. At physiologically high shear rates (1000 to 10,000 s^−1^), a two-stage aggregation process is observed, where unstable clusters of discoid platelets facilitated by membrane tethers and GPIbα-VWF A1 become converted to stable aggregates through platelet activation and mechanotransduction, forming irreversible $\alpha_{IIb}\beta_{3}$-fibrinogen bonds [6,108]. At pathologically high shear conditions (> 10,000 s^−1^), platelet aggregation can be initiated independently of platelet activation, with VWF-GPIbα-VWF bonds being the sole mediator [6,7,37,108]. At these elevated shear rates, platelets change their shape to a smooth, spherical shape [3,6]. Overall, the increase in shear rate leads to more VWF-mediated binding and less platelet activation-induced binding.

Multiscale computational models have been developed to understand platelet aggregation in all three shear regimes. Below, we highlight a few most recent studies for the three shear regimes as indicated in Figure 4a.

For low shear regimes (100 to 1000 s^−1^), Flamm et al. [110] developed a patient-specific model for platelet aggregation in flow. A neural network (NN) model of calcium regulation was trained on patient data and integrated into the model to provide a donor-specific prediction of platelet response in flow. Gupta et al. [111,112] developed a multiscale model combining dissipative particle dynamics and coarse-grained molecular dynamics to simulate the aggregation of free-flowing platelets, where the aggregation is driven by the interaction of $\alpha_{IIb}\beta_{3}$ receptors and fibrinogen (Figure 4b). Generally, these models neglect morphological changes such as activated platelet filopodia formation, which could enhance the aggregation through increased contact area as reported in Maxwell et al. [108].

For the physiologically high shear regime (1000 to 10,000 s^−1^), Fogelson and Guy [113] studied platelet aggregation at a shear rate of 1750 s^−1^ using immersed-boundary type methods. Using this approach, they observe platelets accumulate as mural aggregates after agonist-induced platelet activation. Mori et al. [114] developed a Stokesian Dynamics model to simulate platelet binding with fibrinogen and VWF concurrently under a shear rate of 5000 s^−1^. They found both $\alpha_{IIb}\beta_{3}$-fibrinogen and GPIbα-VWF A1 bonds are necessary to form stable aggregates in this shear regime. Shankar et al. applied a multiscale computational method to simulate thrombus growth across a shear range of 100∼8000 s^−1^ [115], showing the necessity of considering the VWF-mediated platelet aggregation to form occlusion under high shear (Figure 4c). In these models, the receptor–ligand and their molecular kinetics were not modeled directly; instead, adhesion was provided by elastic links between immersed boundary points both on the platelet and the wall.

For the pathologically high shear regime (>10,000 s^−1^) relevant to acute occlusive arterial thrombosis, Liu et al. [3,7] recently developed a multiscale computational model that integrates VWF multimers, inactivated platelets, and GPIbα-VWF A1 binding kinetics based on experimental measurements to unravel the dynamic process of shear-induced platelet aggregation, shown in Figure 4d. Their model predicts that the aggregation process can occur in less than 10 milliseconds, potentially explaining how billions of platelets can be captured in less than 10 min when they pass through arterial stenosis to form an occlusive thrombus. Their model also predicts that loose aggregates can form with ultra-large VWF under high shear to potentially form a porous thrombus. Recently, Du et al. [116], Du and Fogelson [117] developed a continuum model to study occlusive arterial thrombosis formation in a stenosis. Their model confirmed that an ultra-high binding rate and relative porous thrombus are needed in order to form occlusive high-shear thrombi.

## 6. Further Discussion and Outlook

### 6.1. Multiscale Computation as a Tool to Bridge the Gap

**Figure 5:**
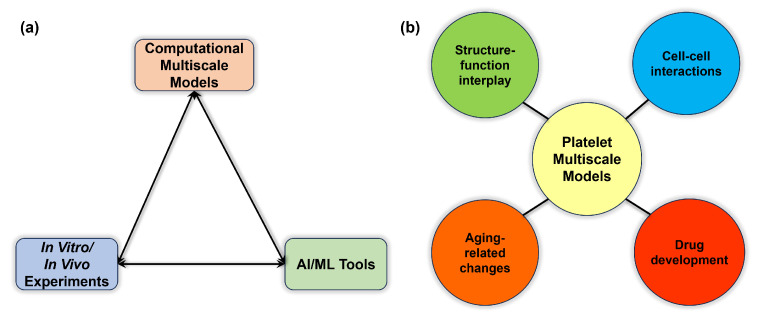
(**a**) Successful computational multiscale models are increasingly integrated with both experimental validation and AI/ML tools. (**b**) Emerging applications of platelet-based multiscale models of thrombosis include intraplatelet structure-function interdependence, interaction with other cell types, effects of aging on platelet properties and function, and antiplatelet drug development).

Biological phenomena such as thrombosis, as described in previous sections, involve many molecular and cellular processes occurring concurrently and synergistically. Due to the multiscale nature, many events are hard to observe simultaneously and directly using existing in vitro or in vivo techniques. As a result, there is a lack of integrated understanding of how individual scale events interact to regulate a full biological process. In these cases, multiscale models are viable tools and sometimes seem to be the only avenue toward a quantitative understanding of the process to unfold unrecognized biological or physical mechanisms. Furthermore, due to the fast-moving platelets and molecules in blood flows, one cannot easily discern the interaction of aggregating platelets, particularly if the interaction occurs in the line of sight of the live microscope. It is important to note that aspects of these models should still be challenged by experimental observations in terms of consistency and correlation with existing experimental data that might be available at a disparate scale. Recent advances in high-performance computing (HPC) and artificial intelligence (AI) are expected to enable data assimilation of experimental data and multiscale computational data from various fidelities and modalities to allow a more comprehensive multiscale understanding of thrombosis processes (Figure 5a).

### 6.2. Developing a Multiscale Model: A Note of Caution

Multiscale models provide excellent avenues for elucidating mechanisms and the underlying mechanobiology involved in how platelets respond to their surrounding flow environment. The examples presented in this review demonstrate that multiscale models are capable of simulating intra- and extra-platelet biological events over large spatial and temporal scales, and as shown in the previous section, can even describe or predict interactions that are difficult to discern experimentally. However, there are a few considerations in determining whether multiscale modeling is appropriate for the phenomena under observation and if the chosen approach will yield credible results. On one hand, most modeling techniques, despite their sophistication, use a simplified or reductionist approach to focus on a few important parameters of relevance to the pathophysiology (i.e., flow conditions, agonists, receptor–ligand interactions, simplified platelet structure/geometry, etc.). Therefore, a thorough understanding of the pathophysiology is needed to make sure the model reflects the least necessary conditions for certain biological processes. Alternatively, the model can be treated as a hypothesis generator to check whether certain conditions are needed while predicting experimentally consistent results. On the other hand, many computational models are limited to spatial and temporal scales due to the computational cost. As a result, different multiscale techniques may need to be coupled or used synergistically to obtain a full-scale understanding of the biological process. Notably, recent advances in AI have witnessed efforts to use machine learning tools to bridge scales more efficiently [118].

### 6.3. Potential New Directions

Despite the remarkable advances in the modeling of flow-mediated thrombosis, significant gaps remain in our understanding of the role of platelet biorheology and mechanobiology in this process. In the previous sections, we presented an overview of the different states and processes platelets undergo in response to their surrounding flow environment. We highlighted some multiscale models that describe these platelet processes. We now present a non-exhaustive array of opportunities for further study of previously unmodeled platelet events linked with thrombosis (Figure 5b).

First, platelet response to external stimuli involves both biochemical changes, informed by receptor–ligand interactions and signaling pathways, as well as structural modifications, either through a direct response to external forces or via downstream effects of signaling pathway-informed intraplatelet changes. Most multiscale models have addressed select receptor–ligand interactions, while only a handful addressed biochemical changes (i.e., agonist-initiated activation or agonist release) or structural platelet deformation. Very few models currently integrate both structural changes and biochemical signaling pathways [115]. Such a model may enable a more accurate representation of the platelet’s structure-function interplay in thrombus initiation and the growing clot.

Second, the development of a stable clot necessitates platelet interactions with other cells, including red blood cells—which not only enhance platelet margination and encourage adhesion, but also modulate platelet reactivity, alter fibrin structure, and are entrapped in growing clots [119]—and activated endothelial cells, which serve as the substrate for the clot via expressed VWF and P-selectin [120]. The inclusion of these cells realistically represents the cellular systems involved in thrombosis.

Third, there is a growing understanding of the synergy between thrombosis and inflammation, particularly underscored during atherosclerosis, and the interaction between platelets and leukocytes playing a central role in the progression of thrombosis [121]. Using multiscale models allows elucidation of understudied pro-inflammatory mechanisms involved in thrombosis (“immunothrombosis”) leading to cardiovascular disease, which has gained additional attention since the outbreak of COVID-19 [122].

Fourth, arterial and venous thromboembolism [123] is more prevalent in older adults, and this is due in part to age-related changes within the platelet and its surrounding environment, as previously described. The platelets undergo both functional and structural changes, and these are not addressed in current multiscale models. The adaptation of modeling approaches to account for both age-related structural and biochemical changes allows for a better understanding of the thrombotic risks associated with aging.

Lastly, one of the main motivations behind the development of multiscale approaches is the development of drug therapy [124], and particularly for thrombosis associated with cardiovascular diseases and devices. Current multiscale models of thrombosis have not directly simulated the effect of drug therapy. Rather, the inhibitory effects of drugs are inferred in the model (i.e., Shankar et al. [115]). Increased computational power and integration with ML tools may make the introduction of drug molecules and their requisite interactions with the platelet feasible.

## 7. Summary

Thrombosis is a complex biorheological and mechanobiological process spanning multiple spatial and temporal scales. Under pathological flow conditions, the platelet marginates towards an injured vessel or device wall, with which it adheres through receptor–ligand interactions. The thrombus grows as flowing platelets aggregate and become captured at the wall. During these phases of thrombosis, platelets encounter molecular ligands (such as VWF) in the presence of fluid shear forces. Platelets may also activate through a mechanotransduction process given sufficient time under specific stress levels. Further complexity arises from the effect of aging on platelet mechanobiology. While these phenomena have been extensively studied in vitro and in vivo, computational biology approaches have become more efficient at probing the intricacies of thrombosis and elucidating the link between molecular-level kinetics and platelet mechanotransductive mechanisms in a growing clot, with advances in replicating platelet mechanobiology. The growing role of artificial intelligence and its integration with computational biology holds the promise of enhancing both benchtop and computational approaches for characterizing and predicting multiscale thrombosis events as a paradigm of digital medicine.

## Figures and Tables

**Table 1:** Hemodynamic parameters in blood vessels and devices (adapted from [12]. LAD, left anterior descending artery; LCA, left coronary artery; RCA, right coronary artery. ^a^ Difficult to define in capillaries; ^b^ Higher shear rates are due to recirculation zones; ^c^ Flow can be turbulent; ^d^ Flow is turbulent; ^e^ Lower shear rates found in valve pocket; ^f^ Higher shear rates lead to acquired von Willebrand disease; ^g^ All shear rates and stresses are on the wall, except for these conditions.

Vessel/Condition	Shear Rate (s^−1^)	Shear Stress (dyne/cm^2^)
Normal physiology		
Ascending aorta	300	2–10
Large arteries	300–800	10–30
Coronary artery (LCA/RCA)	300–1500	10–60
Carotid artery	250	10
Arterioles	500–1600	20–60
Capillary	200–2000	high ^a^
Postcapillary venules	50–200	1–2
Veins	20–2000	0.8–8
Disease conditions		
Coronary stenosis (LAD)	5000–100,000 ^b^	-
Carotid stenosis	-	40–360
Aortic coarctation ^g^	-	140–>1000 ^c^
Atrioventricular fistula ^g^	-	100–1000 ^d^
Deep vein thrombosis ^g^	0–200 ^e^	
Prosthetic devices		
Mechanical heart valves ^g^	-	up to 6000
Left ventricular assist devices ^g^	5000–>100,000 ^f^	0–>1000
